# Case of Compound Fracture of the Skull—Trephining—Death

**Published:** 1839-04-20

**Authors:** 


*Case of Compound Fracture of the Skull—Trephining—Death.*

F. G., æt. sixteen, admitted 3½ P. M. of the 8th of March, 1839, having been injured by a brickbat at 1 P. M. of the same day. A physician, who saw him immediately after the accident, stated that he was at that time perfectly sensible, asked for water, and appeared conscious of all that passed around him. When brought to the hospital, he was almost entirely insensible, though, upon calling him by name, he would open his eyes, yet could not answer questions, and very soon relapsed into insensibility. The pulse was slow, and rather feeble; the pupils dilated, and but slightly sensible to light; the skin warm. There was an external wound extending from half an inch above the middle of the left eyebrow, to an inch above the outer end of the right brow, with a fracture and depression of the bone beneath. The depression was a half or three quarters of an inch in depth, and was caused by three transverse fractures, forming two large fragments, the edges of the central fracture being the parts depressed, thus causing a triangular depression, the point of which rested upon the dura mater. It being impossible to introduce an elevator under the bone, a small trephiné was applied on the sound part to the right, after which the lower fragment was raised to its level. The upper fragment could not be raised, however, until the lower one was entirely removed, as it was bound down by this latter. The boy now became sensible, answered questions, and asked for drink.

9*th*.—Morning. Passed a good night; quite sensible; pupils natural; skin somewhat hot; passed his water; pulse being accelerated, he was bled ℥vi. and cold applied to the head. ℞. Mist. Neutr. ℥vi; Ant. Tatr. gr. j. solut. ℥ss; q. h. h. ℞. Calomel gr. ss. every three hours. Barley water as diet.

Evening. Skin hot; pulse 120; very restless; ℥v. of blood to be taken by cups from the neck, and a drachm each of magnesia and salts to be given. Died at 5 A. M. of the next day, without convulsions.

After death it was found that the fracture and depression involved the inner wall of the upper part of the frontal sinus. A clot of blood of about an inch square was lying upon the dura mater below the seat of fracture. Numerous spicula and laminæ of bone were adherent to and piercing the dura mater, which was roughened and covered with a thick layer of lymph. The arachnoid and pia mater were minutely injected; several masses of coagulated blood about the size of cherry stones were found effused into the substance of the brain, both right and left hemispheres, immediately beneath the contusion. The substance of the brain in general was moist but firm. Lateral ventricles did not contain more fluid than natural.

This case is reported more as being an instance of the extent to which the brain may be injured without influencing immediately its operations, than as useful in a practical point of view. Though the brain was here compressed to a considerable extent, (the upper fragment being above the frontal sinus,) yet for about two hours after the accident he was perfectly sensible, answered questions, mentioned how he was engaged at the time of the accident, and could distinguish colours.

